# Intolerance of uncertainty and suicidal ideation among Chinese college students: the mediating role of non-suicidal self-injury and the stage-specific moderating roles of perceived social support

**DOI:** 10.3389/fpsyg.2026.1796412

**Published:** 2026-03-16

**Authors:** Xuefei Li, Shilei Zhang, Danrui Chen

**Affiliations:** Psychological Counseling Centre, Chang' an University, Xi' an, China

**Keywords:** intolerance of uncertainty, moderated mediation, non-suicidal self-injury, perceived social support, suicidal ideation

## Abstract

**Introduction:**

Intolerance of uncertainty (IU) is increasingly recognized as a transdiagnostic risk factor for suicide-related outcomes. However, the mechanisms linking IU to suicidal ideation remain insufficiently understood.

**Objectives:**

This study aimed to examine the association between IU and suicidal ideation, particularly regarding the role of non-suicidal self-injury (NSSI) and the protective roles of perceived social support from different sources, including family and friend support.

**Methods:**

A cross-sectional survey was conducted among 2, 167 Chinese college students (68.6% females; *M*age = 18.91years, *SD* = 1.31). Participants completed measures of IU, NSSI, suicidal ideation, and perceived family and friend support.

**Results:**

Results indicated: (1) IU was positively associated with both NSSI and suicidal ideation. (2) NSSI partially mediated the association between IU and suicidal ideation. (3) Perceived family support and friend support both moderated the direct association between IU and suicidal ideation, such that higher levels of support attenuated this direct association. (4) Regarding the indirect pathway, friend support moderated the association between IU and NSSI, whereas family support moderated the association between NSSI and suicidal ideation.

**Conclusion:**

The present findings clarify a cognitive–behavioral pathway through which IU contributes to suicidal ideation and highlight perceived social support as a key protective resource that buffers this risk. By revealing differentiated roles of family and friend support across pathways, the study advances understanding of how stage-specific protective roles of perceived social support across the associations among IU, NSSI, and suicidal ideation.

## 1 Introduction

Suicidal ideation, defined as thoughts of killing oneself (Nock et al., 2010), is a critical indicator of suicide risk and a key target for early prevention efforts (Park et al., 2017; Shen et al., 2024a; Udupa et al., 2023). Prior studies have indicated that suicidal ideation is relatively prevalent with lifetime prevalence estimates standing at 17.8 % (Chen et al., 2025; Uddin et al., 2019), which is particularly salient during emerging adulthood (Asante and Atorkey, 2023; Hu et al., 2025). Given its high prevalence and serious outcomes, elucidating the psychological processes that underlie suicidal ideation among university students, therefore, remains an important research priority.

Emerging adulthood, specifically the university period, represents a developmental period marked by heightened instability and rapid change, particularly in relation to academic demands, career trajectories, and future life planning (Barlett et al., 2020; Fassett-Carman et al., 2023). During this stage, individuals are required to make consequential decisions in the absence of clear or stable reference points, rendering tolerance for uncertainty a central psychological capacity (Bottesi, 2024; Jankowiak et al., 2025). For Chinese undergraduates, this capacity is further emphasized by strong societal expectations and a cultural emphasis on certainty and stability (Delvecchio et al., 2023; Zhou et al., 2023). Thus, this study adopts an integrated risk–protection framework to examine how a cognitive vulnerability (intolerance of uncertainty) relates to suicidal ideation through a behavioral risk factor (non-suicidal self-injury), and how this pathway may be moderated by a protective interpersonal resource (perceived social support), in a sample of Chinese undergraduates.

### 1.1 Intolerance of uncertainty

Intolerance of uncertainty (IU) refers to a cognitive bias whereby uncertain situations are perceived as stressful, threatening, and unacceptable (Carleton et al., 2007). Accumulating evidence suggests that IU is a transdiagnostic vulnerability associated with negative emotional responses and maladaptive coping (Lauriola et al., 2018; Mahoney and McEvoy, 2012).

According to the Stress–Diathesis model of suicide, stable cognitive vulnerabilities, such as IU, may interact with stressors to increase suicide risks (Mann et al., 1998). Individuals with high IU are particularly sensitive to uncertainty-related stress, tending to interpret ambiguous or uncontrollable situations as overwhelming and difficult to tolerate (Yao et al., 2023). Such cognitive bias may, in turn, heighten vulnerability to suicidal ideation. Empirical evidence provides support for this perspective. For instance, Ciarrochi et al. (2005) found that IU was positively associated with suicidal ideation among undergraduates. Subsequent studies have similarly shown that higher IU is associated with increased suicidal risks, both in clinical and non-clinical populations (Sen and Yildizhan, 2020; Zhou et al., 2023). However, most of this evidence focuses primarily on the direct link between IU and suicidal ideation, offering limited insight into the psychological or behavioral processes through which IU confers suicide risk. Accordingly, the processes through which IU links to suicidal ideation require further clarification.

### 1.2 Non-suicidal self-injury as a mediating role

Non-suicidal self-injury (NSSI), defined as deliberate self-harm without suicidal intent (Nock, 2010), is a critical proximal risk factor for suicidal ideation (Hasking et al., 2019; Muehlenkamp et al., 2023). According to the Interpersonal Models of Suicide, repeated engagement in NSSI may increase cognitive accessibility to self-harm–related thoughts, thereby elevating vulnerability to suicidal ideation (Joiner, 2005; Victor and Klonsky, 2018). Extensive research has also shown that individuals who engage in NSSI are at substantially elevated risk for subsequent suicidal ideation, even after controlling for psychiatric symptoms such as depression and anxiety (Hamza et al., 2012; Nock et al., 2006; Ribeiro et al., 2016).

Importantly, individuals with high IU may be particularly vulnerable to engaging in NSSI. From a functional perspective, NSSI is commonly conceptualized as an immediate, controllable behavior for regulating aversive internal states (Klonsky, 2007; Nock, 2010). Individuals high in IU, who experience uncertainty as aversive and intolerable, may therefore be more inclined to adopt NSSI (Yao et al., 2023). Specifically, NSSI may serve as a maladaptive strategy to replace an amorphous, uncontrollable threat (i.e., uncertainty) with a tangible, predictable, and self-controlled physical sensation (i.e., pain). Emerging evidence suggested that higher IU were associated with increased engagement in self-harm-related behaviors (e.g., Maloret and Scott, 2018). Taken together, NSSI may serve as a key behavioral pathway through which IU may be translated into elevated suicidal ideation.

### 1.3 Perceived social support as a protective factor

Perceived social support refers to an individual's subjective evaluation of the availability and adequacy of support from significant others, such as family members and friends (Zimet et al., 1988). From a positive psychology perspective, perceived social support represents a core psychological resource that contributes to individuals' adaptive functioning, motivational regulation, and resilience in the face of uncertainty. Rather than serving solely as a buffer against risk, social support may play distinct functional roles at different stages of psychological vulnerability, shaping how individuals interpret uncertainty and regulate distress-related behaviors. Substantial research has consistently identified perceived social support as a protective factor against suicidal ideation and NSSI among adolescents and young adults (Borschmann and Moran, 2022; Che et al., 2022; Zhang et al., 2022). Beyond its direct protective role, accumulating evidence suggests that perceived social support may also buffer the impact of cognitive vulnerabilities like IU on mental problems (Wang et al., 2025).

From a theoretical perspective, the motivational–volitional model (IMV; O'Connor, 2011) emphasizes that social and interpersonal factors are conceptualized as protective resources that may mitigate the impact of psychological risk factors on suicide-related outcomes. Social support may reduce psychological distress by enhancing emotional security, promoting adaptive coping, and increasing perceived belongingness and meaning in life (Hollingsworth et al., 2018; Guo et al., 2025).

Most existing studies have focused on overall levels of perceived social support, with relatively limited attention to the potentially distinct roles of different support sources (Zhu Y. et al., 2021; Zhu Z. et al., 2021). Family support and friend support may represent qualitatively different interpersonal resources, serving different functions during emerging adulthood (Lee et al., 2018, 2020a,b), which represents a period marked by heightened uncertainty, identity exploration, and increasing reliance on peer relationships. University students tend to shift their primary sources of emotional support from family to peers as they navigate academic, interpersonal, and future-related uncertainties (Arnett, 2000, 2004). Empirical studies have similarly shown that friends play a particularly salient role in daily emotional regulation and stress coping during this developmental stage, whereas family support often becomes more prominent in contexts of more severe psychological distress (Furman and Buhrmester, 1992). These processes may be further shaped by cultural context. In collectivist societies such as China, family relationships are deeply embedded in moral obligation, interdependence, and long-term responsibility, which may render family support particularly salient at more severe stages of suicide risk (Chu et al., 2010; Zhang and Jia, 2011).

Accordingly, differentiating perceived family support and friend support may provide a more nuanced understanding of how interpersonal resources shape both direct and indirect pathways linking cognitive vulnerabilities to suicidal ideation. However, empirical evidence regarding the stage-specific roles of different sources of social support remains limited. Accordingly, the present study examines whether family and friend support differently moderate associations among IU, NSSI, and suicidal ideation, without pre-specifying their specific moderation effects.

### 1.4 The present study

Despite growing evidence linking IU to suicide-related outcomes, several limitations remain in the existing literature. First, prior research has largely focused on the direct association between IU and suicidal ideation, with relatively limited attention to the potential mechanisms underlying the direct association. Second, although perceived social support is widely recognized as a protective factor, most studies have treated social support as a unidimensional construct, while less is known about whether different sources of support—specifically family support and friend support—play distinct roles in shaping both the direct and indirect associations between cognitive vulnerabilities and suicidal ideation. Third, undergraduates routinely face multiple sources of uncertainty (e.g., academic demands and future career prospects; Fassett-Carman et al., 2023), which may heighten sensitivity to uncertainty and vulnerability to maladaptive coping and suicide-related outcomes.

**Figure 1:**
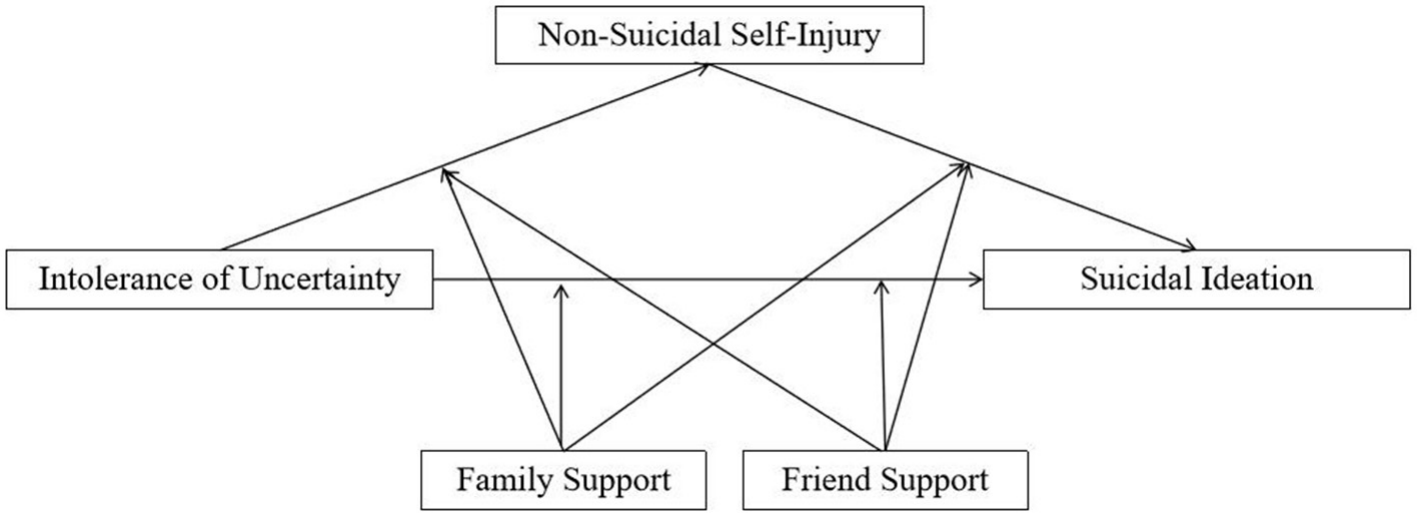
Hypothesized moderated mediation model from intolerance of uncertainty to suicidal ideation.

However, empirical studies focusing on IU within undergraduates remain limited. To address these gaps, the present study proposes a moderated mediation framework (as shown in Figure 1) to examine the association between IU and suicidal ideation among undergraduates. Specifically, we examine (a) whether NSSI mediates the relationship between IU and suicidal ideation, and (b) whether perceived family support and friend support moderate the direct effect and the indirect effect. Given the limited prior evidence regarding the stage-specific roles of different sources of perceived social support, the present study did not propose specific a priori hypotheses regarding whether family or friend support would operate at particular stages of the IU–NSSI–suicidal ideation pathway. Instead, we adopted a theoretically informed but exploratory approach to examine whether different sources of social support might differently moderate associations across stages.

## 2 Methods

### 2.1 Participants and procedure

This study employed a cross-sectional survey design. Participants were 2, 167 undergraduate students recruited from a university in Guangdong Province, China. The average age of participants was 18.91 years (*SD* = 1.31), and 68.6% of the sample were female. Data were collected online via the professional platform *Wenjuanxing*. Participation was voluntary and anonymous. All participants provided informed consent after being informed of the study's purpose. Ethical approval was obtained from the institutional ethics committee of the authors' university.

### 2.2 Measures

#### 2.2.1 Intolerance of uncertainty (IU)

Measured using the Chinese version of the Intolerance of Uncertainty Scale−12 (IUS-12; Carleton et al., 2007; Yao et al., 2023). The scale consists of 12 items assessing individuals' reactions to uncertain situations over the past six months. Participants rated each item on a 5-point Likert scale ranging from 1 (strongly disagree) to 5 (strongly agree). Higher total scores indicated greater IU. Previous studies have supported the reliability and validity of the scale (Yao et al., 2023; Zhou et al., 2023). In the present study, the scale demonstrated excellent internal consistency (Cronbach's α = 0.942).

#### 2.2.2 Non-suicidal self-injury (NSSI)

Assessed using a 12-item scale adapted from the Deliberate Self-Harm Inventory (DSHI; Gratz, 2001). Participants were asked to indicate whether they had engaged in specific self-injurious behaviors without suicidal intent during the past 6 months. Responses were recorded on a 6-point scale ranging from 0 (never) to 5 (five times or more), with higher scores reflecting more frequent engagement in NSSI behaviors. Prior research has supported the reliability and validity of this measure in Chinese adolescent and young adult samples (You et al., 2015). In the present study, the scale demonstrated good internal consistency, with a Cronbach's α of 0.873.

#### 2.2.3 Suicidal ideation

Assessed via a single self-report item. Participants were asked to indicate how often they had experienced suicidal thoughts during the past 6 months. Responses were recorded on a 6-point ordinal scale: 0 (never), 1 (once), 2 (twice), 3 (three times), 4 (four times), and 5 (five times or more). Higher scores reflected more frequent suicidal ideation.

#### 2.2.4 Perceived social support

Measured using the family support and friend support subscales of the Chinese Perceived Social Support Scale (PSSS; Jiang, 2001). Each subscale consists of four items assessing perceived support from family members and friends, respectively. Participants responded to each item on a 7-point Likert scale ranging from 1 (very strongly disagree) to 7 (very strongly agree), with higher scores indicating greater perceived social support. Previous studies have demonstrated good reliability and validity of the scale in Chinese populations (Wu et al., 2022). In the present study, both subscales demonstrated excellent internal consistency (family support: Cronbach's α = 0.938; friend support: Cronbach's α = 0.956).

### 2.3 Statistical analysis

Analyses were conducted using SPSS version 21 with the PROCESS macro (version 3.2; Hayes, 2018). Participants reported their age and gender, with gender coded as 0 = female and 1 = male; both variables were included as covariates in model testing. Prior to hypothesis testing, the distributional properties of all study variables were examined. Among these variables, only NSSI showed pronounced non-normality, with skewness (7.226) and kurtosis (70.208) exceeding commonly accepted thresholds (skewness < 3, kurtosis < 10; Kline, 2016). Given the extreme positive skewness of NSSI frequency, a logarithmic transformation was applied to improve normality and stabilize variance. Similar transformations have been widely used in prior NSSI research to address distributional violations and enhance the robustness of regression-based analyses (Hasking et al., 2019). While transformed coefficients reflect changes on a logarithmic scale, this approach improves comparability and statistical validity. Therefore, a logarithmic transformation was applied to the NSSI scores to improve normality (after transformation, skewness = 2.908, kurtosis = 7.515). Pearson correlation analyses were subsequently conducted to examine bivariate associations among the study variables.

Next, PROCESS Models 6 and 76 were employed to examine the serial mediation and moderated mediation effects. Indirect effects were tested using bias-corrected bootstrapping with 10,000 resamples. Effects were considered statistically significant if the 95% confidence interval did not include zero. For significant interaction effects, simple slope analyses were conducted at low (−1 SD), mean, and high (+1 SD) levels of the moderator. Age and gender were included as covariates in all models, and all study variables were standardized (z-scored) prior to analysis.

## 3 Results

### 3.1 Preliminary analyses

Descriptive statistics and correlation coefficients for all study variables are presented in Table 1. IU was positively correlated with NSSI and suicidal ideation. Perceived family support and friend support were negatively correlated with IU, NSSI and suicidal ideation. NSSI was positively associated with suicidal ideation.

**Table 1:** Means, standard deviations, and correlations among variables (*N*= 2,167).

**Variables**	**1**	**2**	**3**	**4**	**5**
1 Intolerance of Uncertainty (IU)					
2 Non-Suicidal Self-Injury (NSSI)	0.273				
3 Suicidal ideation	0.442	0.419			
4 Family support	−0.217	−0.137	−0.269		
5 Friend support	−0.169	−0.123	−0.207	0.745	
Mean	26.300	0.865	0.470	12.651	12.976
SD	11.044	3.728	1.261	4.395	4.224

All correlations in this table were statistically significant.

### 3.2 Mediation analysis

**Figure 2:**
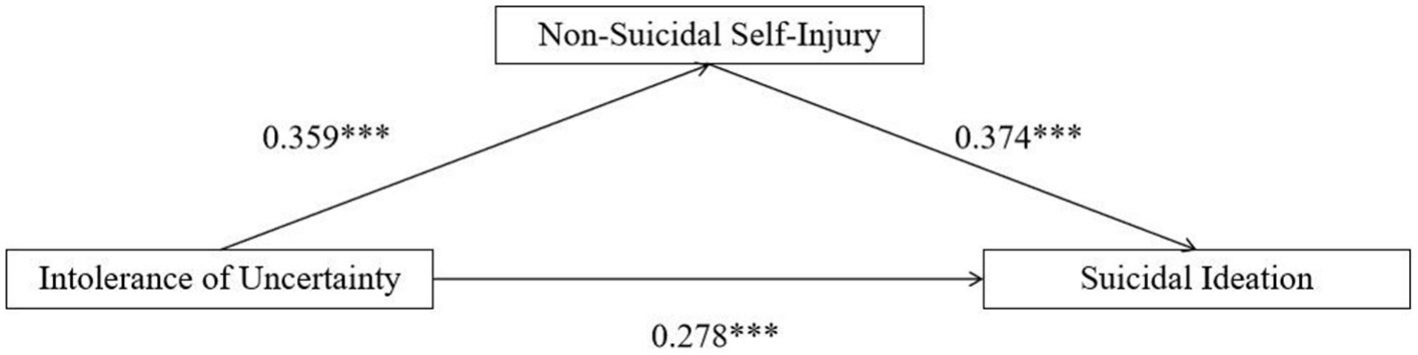
Standardized path coefficients for the mediation model of intolerance of uncertainty, non-suicidal self-injury, and suicidal ideation. ***p < 0.001.

We first examined whether NSSI mediated the association between IU and suicidal ideation. As shown in Figure 2, results showed that IU was significantly and positively associated with NSSI [β = 0.359, SE = 0.021, *p* < 0.001, 95% CI (0.318, 0.400)]. NSSI was also significantly associated with higher levels of suicidal ideation [β = 0.374, SE = 0.019, *p* < 0.001, 95% CI (0.337, 0.412)]. The direct effect of IU on suicidal ideation remained significant after including NSSI in the model [β = 0.278, SE = 0.020, *p* < 0.001, 95% CI (0.239, 0.316)].

Bootstrapping analyses with 10,000 resamples indicated that the indirect effect of IU on suicidal ideation via NSSI was significant [*indirect effect* = 0.134, 95% CI (0.103, 0.169), accounting for 32.598% of the total effect], supporting the mediating role of NSSI. Taken together, these results suggest that NSSI partially mediates the association between IU and suicidal ideation, with IU exerting both a direct effect and an indirect effect through NSSI.

### 3.3 Moderation of the direct path

**Figure 3:**
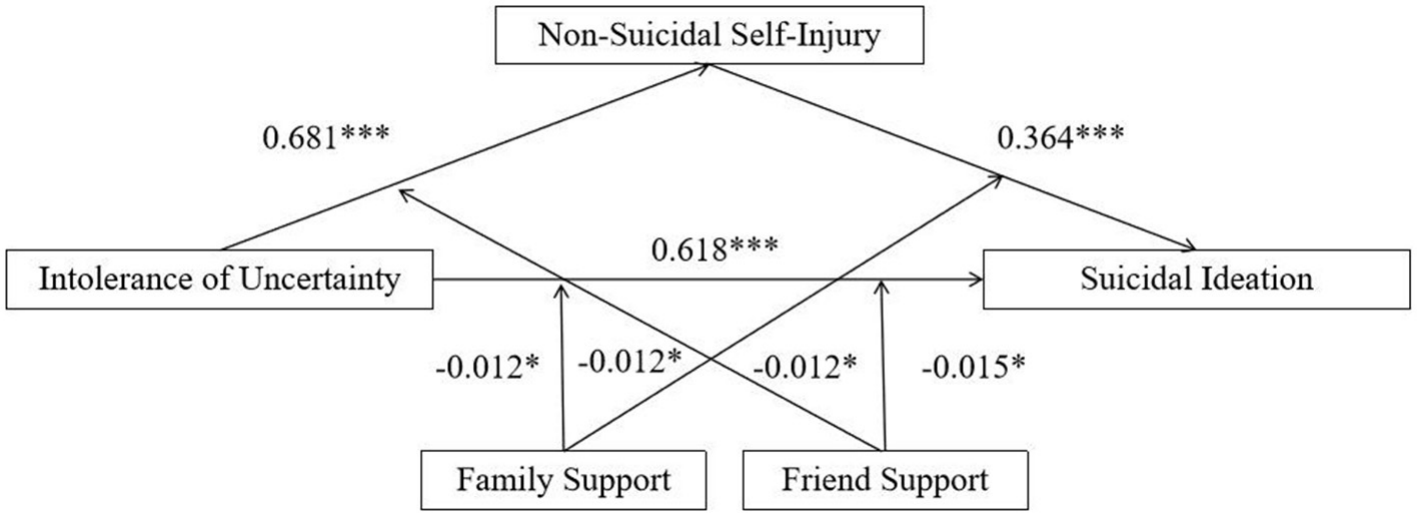
Standardized path coefficients for the proposed moderated mediation model. *p < 0.05, ***p < 0.001.

Figure 3 showed the standardized path coefficients for the proposed moderated serial mediation model. Results indicated that both perceived family support and friend support moderated the direct association between IU and suicidal ideation. Specifically, the interaction between IU and family support significantly predicted suicidal ideation [β = −0.050, SE = 0.025, *p* < 0.05, 95% CI (−0.100, −0.001)], and the interaction between IU and friend support was also significant [β = −0.064, SE =0.025, *p* < 0.05, 95% CI (−0.112, −0.015)].

**Figure 4:**
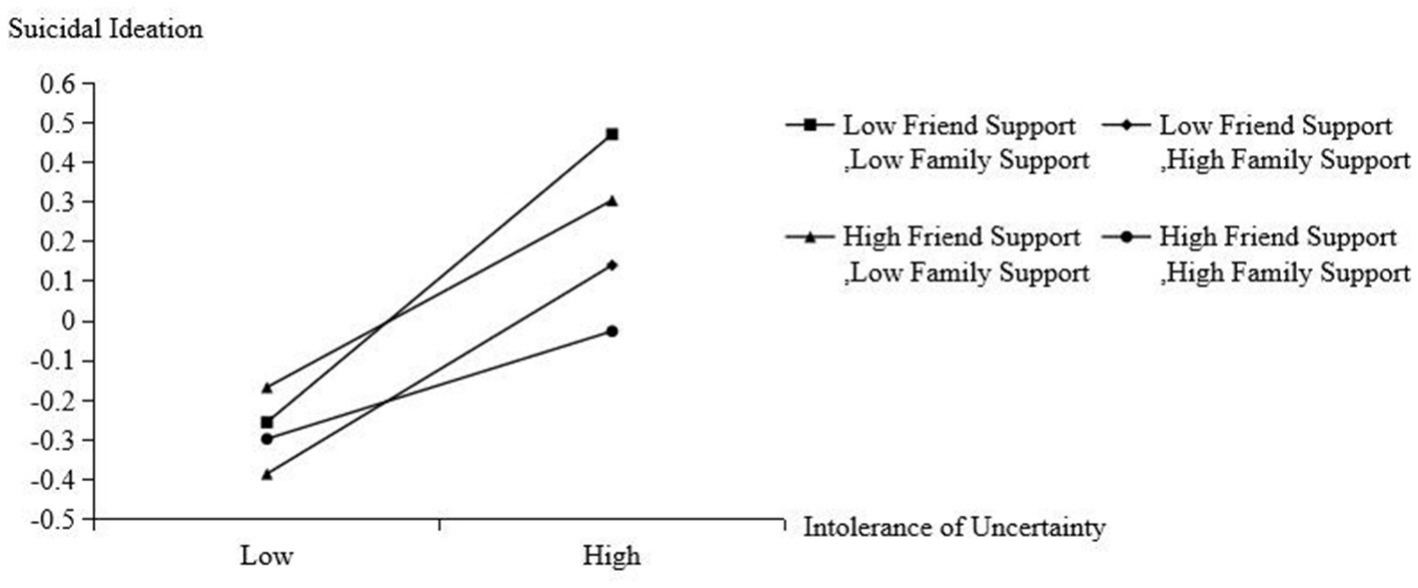
Plot of friend support and family support as moderator between intolerance of uncertainty and suicidal ideation. Low = −1 SD; Mean = 0 SD.

Conditional direct effects analyses further illustrated these interaction effects (see Table 2 and Figure 4). The associations between IU and suicidal ideation were all significant across all combinations of family and friend support, but decreased as levels of perceived social support increased, indicating a buffering effect of social support on the association between IU and suicidal ideation. Specifically, the strongest effect was observed when both family support and friend support were low [*b* = 0.363, SE = 0.026, *p* < 0.001, 95% CI (0.313, 0.414)], whereas the weakest effect occurred when both supports were high [*b* = 0.136, SE = 0.028, *p* < 0.001, 95% CI (0.082, 0. 190)].

**Table 2:** Conditional direct effects of IU on suicidal ideation at different levels of social support.

**Family support**	**Friend support**	**Effect**	**SE**	***t***	***p***	**LLCI**	**ULCI**
Low	Low	0.363	0.026	14.091	< 0.001	0.313	0.414
Low	Mean	0.300	0.031	9.569	< 0.001	0.238	0.361
Low	High	0.236	0.050	4.697	< 0.001	0.138	0.335
Mean	Low	0.313	0.031	10.030	< 0.001	0.252	0.375
Mean	Mean	0.250	0.020	12.826	< 0.001	0.212	0.288
Mean	High	0.186	0.032	5.852	< 0.001	0.124	0.248
High	Low	0.263	0.051	5.213	< 0.001	0.164	0.362
High	Mean	0.200	0.032	6.187	< 0.001	0.136	0.263
High	High	0.136	0.028	4.934	< 0.001	0.082	0.190

Low = −1 *SD*; Mean = 0 *SD*; High = +1 *SD*. All effects significant (*ps* < 0.001).

### 3.4 Moderation of the indirect paths

**Figure 5:**
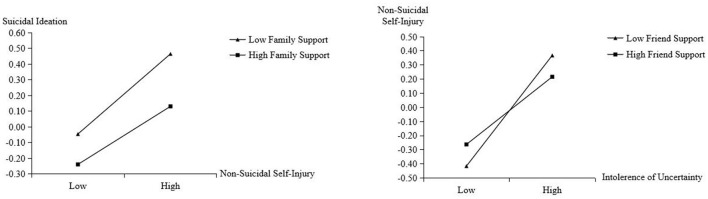
Plot of family support as moderator between non-suicidal self-injury and suicidal ideation **(Left)**, and plot of friend support as moderator between intolerance of uncertainty and non-suicidal self-injury **(Right)**. Low = −1 SD; Mean = 0 SD; High = 1 SD.

Regarding the first-stage path, the interaction between IU and friend support significantly predicted NSSI [β = −0.076, SE = 0.027, *p* < 0.01, 95% CI (−0.129, −0.024)], indicating that friend support moderated the association between IU and NSSI. In contrast, the interaction between IU and family support was not significant [β = −0.051, SE = 0.027, *p* = 0.063, 95% CI (−0.105, 0.003)]. To further interpret the significant interaction, conditional effects analyses were conducted. As shown in Figure 5, IU was positively associated with NSSI when friend support was low [*b* = 0.392, SE = 0.033, *p* < 0.001, 95% CI (0.327, 0.457)]. This association was weaker, though still significant, when friend support was high [*b* = 0.239, SE = 0.025, *p* < 0.001, 95% CI (0.171, 0.308)]. These conditional effects indicate that the positive association between IU and NSSI varies as a function of perceived friend support. Specifically, IU showed a stronger association with NSSI among individuals reporting lower levels of friend support, whereas this association was weaker among those with higher levels of friend support. Although the association remained statistically significant across levels of friend support, the attenuation observed at higher support levels suggests that friend support is associated with reduced strength of the IU–NSSI link.

Regarding the second-stage path, the interaction between NSSI and family support significantly predicted suicidal ideation [β = −0.050, SE = 0.024, *p* < 0.05, 95% CI (−0.100, −0.004)], suggesting that family support moderated the association between NSSI and suicidal ideation. However, the interaction between NSSI and friend support was not significant [β = 0.034, SE = 0.022, *p* = 0.122, 95% CI (−0.009, 0.078)]. As shown in Figure 5, conditional effects analyses indicated that NSSI was positively associated with suicidal ideation when family support was low [*b* = 0.378, SE = 0.027, *p* < 0.001, 95% CI (0.325, 0.431)]. This association was weaker at high levels of family support [*b* = 0.271, SE = 0.037, *p* < 0.001, 95% CI (0.201, 0.346)]. Similarly, the conditional effects for the second-stage path indicate that the association between NSSI and suicidal ideation differed across levels of perceived family support. When family support was low, NSSI was more strongly associated with suicidal ideation, whereas this association was attenuated at higher levels of family support. These results suggest that higher perceived family support is associated with a weaker statistical linkage between NSSI and suicidal ideation.

As shown in Table 3, the indirect effect of IU on suicidal ideation via NSSI was statistically significant across all combinations of family support and friend support. However, the magnitude of the indirect effect systematically decreased as levels of perceived social support increased. Specifically, the strongest indirect effect was observed when both family support and friend support were low [Effect = 0.152, SE = 0.024, 95% CI (0.107, 0.199)]. As either source of support increased, the magnitude of the indirect effect gradually attenuated. When both family support and friend support were at high levels, the indirect effect was substantially weaker [Effect = 0.058, SE = 0.017, 95% CI (0.027, 0.094)]. This pattern indicates that higher levels of perceived social support—particularly when both family and friend support are concurrently high—are associated with a weaker indirect association between IU and suicidal ideation through NSSI.

**Table 3:** Conditional indirect effects at different levels of social support.

**Family support**	**Friend support**	**Effect**	**SE**	**LLCI**	**ULCI**
Low	Low	0.152	0.024	0.107	0.199
Low	Mean	0.139	0.026	0.091	0.193
Low	High	0.120	0.040	0.051	0.204
Mean	Low	0.114	0.025	0.067	0.167
Mean	Mean	0.103	0.015	0.073	0.134
Mean	High	0.086	0.022	0.047	0.132
High	Low	0.082	0.036	0.020	0.161
High	Mean	0.072	0.022	0.033	0.121
High	High	0.058	0.017	0.027	0.094

Low = −1 *SD*; Mean = 0 *SD*; High = +1 *SD*.

## 4 Discussion

The present study aimed to clarify how IU associated to suicidal ideation among undergraduates within a moderated mediation framework. As hypothesized, IU was associated with higher levels of suicidal ideation both directly and indirectly through NSSI. Moreover, perceived social support showed pathway-specific moderating effects, underscoring the importance of distinguishing between different sources of support. Several important findings emerged, extending existing research on cognitive vulnerability, maladaptive coping, and interpersonal protective factors in suicide risk.

### 4.1 IU, NSSI, and suicidal ideation

Consistent with prior research (e.g., Ciarrochi et al., 2005; Zerach and Levi-Belz, 2019), we found a strong positive association between IU and suicidal ideation among undergraduates, indicating that cognitive vulnerability to uncertainty serves as a critical psychological risk factor for suicidal thoughts. Individuals high in IU tend to perceive ambiguous and uncontrollable situations as threatening, which may theoretically intensify feelings of helplessness and increase escape-oriented cognitions, thereby facilitating the activation of suicidal ideation (Mann, 1998; Carleton, 2016).

This process may be particularly salient during the university period, a developmental stage characterized by academic pressures, future-related uncertainty, and identity exploration (Arnett, 2000). When individuals lack the capacity to tolerate and regulate uncertainty, stressors may be experienced as persistent and overwhelming psychological burdens (Hirsh et al., 2012; Mahoney and McEvoy, 2012). By demonstrating this association in a large sample of Chinese undergraduates, the present study extends prior findings underscoring the relevance of IU in non-Western undergraduate populations.

Extending prior work, the present study found that NSSI partially mediated the relationship between IU and suicidal ideation, providing empirical support for a behavioral pathway linking cognitive vulnerability to suicide-related outcomes. Whereas, previous research has primarily explained engagement in NSSI in terms of emotion dysregulation or impulsivity (Gratz and Roemer, 2008; Liu et al., 2017; Shen et al., 2024b), the present findings suggest that uncertainty-related cognitive vulnerability may also increase the likelihood of adopting NSSI as a coping strategy.

The partial mediation of NSSI suggests a “cognitive—behavioral—affective” cascade. For those high in IU, this uncertainty may lead them to engage in NSSI as a form of experiential avoidance strategy—it provides an immediate, concrete focus that terminates the distress associated with IU. However, consistent with the Ideation-to-Action framework, repeated self-harm may lower the psychological barrier to suicide by habituating NSSI and increasing the cognitive salience of self-harm (Klonsky et al., 2021). Importantly, IU retained a significant direct association with suicidal ideation after including NSSI as a mediating variable. This suggests that additional mechanisms—such as rumination, hopelessness, or impaired problem-solving—may also explain the relationship between IU and suicidal ideation, warranting further investigation in future research.

### 4.2 Moderating effects of social support on the direct pathway

The present study found that both family support and friend support buffered the direct association between IU and suicidal ideation, such that the strength of this association decreased as perceived social support increased. The finding is consistent with the social support buffering hypothesis (Cohen and Wills, 1985), which proposes that social support can attenuate the adverse psychological effects of stress-related vulnerabilities. For individuals high in IU, uncertain situations are often experienced as threatening and uncontrollable (Carleton, 2016; Hirsh et al., 2012). Support from family and friends may facilitate cognitive reappraisal of uncertainty, reduce emotional arousal, and enhance perceived coping capacity (Thoits, 2011), thereby weakening the link between IU and suicidal ideation. This finding reinforces the well-established protective role of social support in suicidal risks and extends prior work by demonstrating that social support can attenuate risk arising from uncertainty-related cognitive vulnerability.

### 4.3 Stage-specific roles of social support

A key finding was the distinct, stage-specific moderating roles of different support sources. Friend support moderated the association between IU and NSSI, whereas family support moderated the association between NSSI and suicidal ideation. The stage-specific moderation findings are now explicitly described as exploratory but theoretically interpretable in light of the Integrated Motivational–Volitional (IMV) model (O'Connor, 2011; O'Connor and Kirtley, 2018), rather than as confirmatory evidence of predefined stage mechanisms. This pattern suggests that social support operates in a stage-specific and context-dependent manner during the development of suicide risk, which is consistent with the motivational–volitional model of suicide, which posits that different protective factors may operate at distinct phases of suicide risk development (O'Connor and Kirtley, 2018).

Friend support may play a particularly critical role in the IU-NSSI pathway. For undergraduates, friend relationships are highly accessible and central to daily emotional regulation (Arnett, 2011), acting as the primary “first responders” in their daily lives. Higher levels of friend support may provide immediate emotional validation and social communication (Liu et al., 2022; Olsavsky et al., 2023), thereby reducing the likelihood of resorting to NSSI as a coping strategy (Zhou et al., 2025). This finding is consistent with prior evidence highlighting the protective role of friend support against self-injurious behaviors. In contrast, family support played a more decisive role in the second stage from NSSI to suicidal ideation. When an individual has already engaged in NSSI, they are in a state of heightened clinical risk (Hamza et al., 2019). Within the Chinese cultural context, family relationships are often characterized by strong emotional bonds and long-term obligations, which may enhance feelings of belongingness and responsibility (Chen et al., 2024; Tian et al., 2025). Such support may help constrain the progression from NSSI to suicidal ideation by reinforcing reasons for living and mitigating risks. This result aligns with previous research emphasizing the protective function of family support in contexts of severe psychological distress (Laglaoui Bakhiyi et al., 2017; Tian et al., 2019).

Together, these results underscore the importance of differentiating sources of social support when examining suicide risk processes. Friend support functions as a “front-line” buffer for daily stressors, whereas family support acts as a “last-resort” safety net that reinforces a sense of meaning. Such distinctions provide a more nuanced understanding of how interpersonal resources shape the cognitive vulnerability–behavior–suicidal ideation pathway among undergraduates. Importantly, this stage-specific pattern extends beyond a simple stress-buffering interpretation. Rather than viewing social support solely as attenuating negative outcomes, the present findings suggest that different sources of support may serve distinct regulatory functions across psychological processes. From a positive psychology perspective, this highlights how positive relational resources are mobilized differentially depending on the nature and severity of psychological distress, offering a more nuanced understanding of how psychology resources interact with vulnerability factors.

### 4.4 Limitations and future directions

Several limitations should be acknowledged when interpreting the present findings. First, the cross-sectional design precludes causal inference. Although the proposed moderated mediation model was theoretically grounded, the temporal ordering among intolerance of uncertainty, NSSI, and suicidal ideation cannot be definitively established. Given the cross-sectional nature of the data, temporal precedence among IU, NSSI, and suicidal ideation cannot be established. Alternative explanations, including reciprocal or reverse associations, remain plausible and should be examined using longitudinal or prospective designs in future research.

Second, all variables were assessed using self-report measures, which may introduce common method bias and reporting distortions. Future studies may benefit from incorporating multi-informant data, behavioral indicators, or ecological momentary assessment to capture fluctuations in uncertainty-related cognition, self-injurious behaviors, and suicidal ideation in daily life.

Third, suicidal ideation was assessed with a single-item measure, which, despite its practicality, may lack the reliability and multidimensional sensitivity of established scales, potentially obscuring nuanced relationships. Although single-item measures have been used in large-scale and ethically sensitive studies—particularly among non-clinical populations—to reduce participant burden and distress (e.g., Zhou et al., 2023), such measures may lack the sensitivity to capture nuanced variations in suicidal thoughts. Future studies should employ validated multi-dimensional scales to assess suicidal ideation.

Fourth, the sample consisted of Chinese undergraduates, which may limit the generalizability of the findings to other age groups or cultural contexts. Although this focus strengthens developmentaln specificity, replication across diverse populations is necessary to determine whether the observed pathway-specific moderating effects of social support are universal or context-dependent.

Finally, while the present study focused on NSSI as a key behavioral mediator, other potential mechanisms—such as rumination, hopelessness, or problem-solving deficits—were not examined. Future research could integrate multiple mediators within a longitudinal framework to provide a more comprehensive account of how IU contributes to suicidal ideation.

### 4.5 Implications

From a theoretical perspective, the present study contributes to the literature on suicidal ideation in two main ways. On the one hand, it identifies IU as a salient cognitive vulnerability among undergraduates, extending prior research that has primarily focused on affective dysregulation or personality traits (e.g., Zerach and Levi-Belz, 2019). The findings indicate that uncertainty-related cognitive processing constitutes a distinct pathway through which suicide risk may emerge, thereby broadening theoretical accounts of cognitive risk factors for suicidal ideation. On the other hand, by demonstrating the partial mediating role of NSSI, the study clarifies how cognitive vulnerability may be translated into suicidal ideation through maladaptive coping behaviors, providing empirical support for ideation-to-action frameworks. In addition, the pathway-specific moderating effects of social support further suggest that interpersonal resources operate in differentiated ways across stages of suicide risk, underscoring the value of integrating cognitive and interpersonal perspectives within theoretical models of suicidal ideation (O'Connor and Kirtley, 2018).

From a practical perspective, the findings likewise have clear implications for suicide prevention in university settings. On the one hand, screening for IU may facilitate earlier identification of students at elevated risk for suicidal ideation, particularly in developmental contexts characterized by heightened uncertainty regarding academic and future trajectories. Interventions aimed at enhancing tolerance for uncertainty and promoting adaptive coping strategies may help reduce reliance on NSSI and, in turn, suicidal ideation (Boswell et al., 2013). On the other hand, the differentiated buffering roles of family and friend support highlight the importance of stage-sensitive prevention efforts that leverage peer resources and family involvement to more effectively disrupt the progression from cognitive vulnerability to NSSI and suicidal thoughts. The findings of this study highlight the stage-specific role of social support in the IU–NSSI–suicidal ideation pathway, with friend support moderating the IU–NSSI pathway and family support moderating the NSSI–suicidal ideation pathway. These insights have important clinical implications for intervention and prevention strategies. In university counseling centers, programs aimed at enhancing peer support networks may be especially effective for students who are early in the risk process, particularly those who are experiencing heightened uncertainty but have not yet engaged in self-injury, whereas once NSSI behaviors have emerged, family-based interventions may be more crucial in reducing the escalation from self-injury to suicidal ideation.

## Data Availability

The raw data supporting the conclusions of this article will be made available by the authors, without undue reservation.
